# The Role of a Decision Support System in Back Pain Diagnoses: A Pilot Study

**DOI:** 10.1155/2019/1314028

**Published:** 2019-03-25

**Authors:** Achim Benditz, Florian Faber, Gabriela Wenk, Tina Fuchs, Natalie Salak, Joachim Grifka, Matthias Vogl, Matthias Menke, Petra Jansen

**Affiliations:** ^1^Department of Orthopaedics, University Medical Centre Regensburg, Asklepios Klinikum Bad Abbach, Kaiser-Karl-V.-Allee 3, 93077 Bad Abbach, Germany; ^2^Allianz Private Insurance AG, Königinstraße 28, 80802 Munich, Germany; ^3^Department of Sport Science, University of Regensburg, Universitaetsstraße 31, 93053 Regensburg, Germany

## Abstract

It is the main goal of this study to investigate the concordance of a decision support system and the recommendation of spinal surgeons regarding back pain. 111 patients had to complete the decision support system. Furthermore, their illness was diagnosed by a spinal surgeon. The results showed significant medium relation between the DSS and the diagnosis of the medical doctor. Besides, in almost 50% of the cases the recommendation for the treatment was concordant and overestimation occurred more often than underestimation. The results are discussed in relation to the “symptom checker” literature and the claim of further evaluations.

## 1. Introduction

It is estimated that between 5% and 10% will be suffering from chronic back pain in their lives which leads to high treatment costs [1]. Back pain is also one of the main reasons why people seek health care services once in their lives [2]. In all age groups low back pain is an increasing problem in modern societies. The incidence of low back pain is 60% to 90%, and low back pain is the main cause of working disability in most countries [3–6]. The incidence of neck pain in clinical studies ranges between 10.4% and 71.5%, and the annual prevalence is estimated to vary between 30% and 50% [4, 7–12]. The average annual age-adjusted incidence rates per 100.000 population for cervical radiculopathy are 83.2 and age-specific 202.9 in the age group 50-54 years [13]. Due to these enormous costs for the health insurance, a computerized decision support system (DSS) should be implemented and evaluated. The DSS can be considered as one specific form of a symptom checker. Symptom checkers use computerized algorithms and ask a series of questions about symptoms or require giving them details of symptoms. The kind of algorithms varies but symptom checkers are used by an organisation as the National Health Service, the Mayo Clinic for example. One specific symptom checker as the iTriage is used 50 million times a year [14]. Semigran et al. (2015) evaluated 23 symptom checkers for self-diagnoses and triage [15]. For this evaluation they used 45 standardized patient vignettes. Those 45 vignettes were divided into three subgroups, 15 vignettes for that emergent care was necessary, 15 for that nonemergent care was required, and 15 vignettes where self-care is sufficient. The results showed that the 23 symptom checkers provided the correct diagnoses in 34% of standardized patients and the correct diagnoses under the top 20 diagnoses was given in 58% of the standardized patient evaluations and provided the correct triage advice in 80% of emergent case, 55% of nonemergent cases and 33% of self-care cases. For that, the authors conclude that the symptom checkers have several limitations.

## 2. Aim of the Study

The aim of this pilot study is to evaluate the use of a newly developed decision support system (DSS), which can be considered as a specific symptom checker. In contrast to the study of Semigran et al. (2015), this DSS concentrates on the diagnoses of back pain; however, the result is compared to the examination of the medicine doctors, and not to constructed vignettes. Due to this aim it has to be investigated if there is a relation between the DSS and the medical diagnoses and treatment recommendation.

## 3. Methods

This nonrandomized unblended correlational study included male and female patients with back pain who visited the Department of Orthopaedics of the University Medical Centre Regensburg between August and September 2018. Participation in this study was voluntary. Inclusion criteria were any complaints about back pain. Patients had to speak German language to understand the diagnostic tool. Exclusion criteria were missing consent or patients who were not able to take part. The study was approved by the Ethics Commission of the University of Regensburg (21.08.18, 18-1007-121, EUDAMED CIV-18-02-023086, DRKS DRKS00012467) and carried out in accordance with the approved guidelines of the Helsinki Declaration of 1975. A written informed consent was obtained from all study participants.

### 3.1. Patients

**Figure 1:**
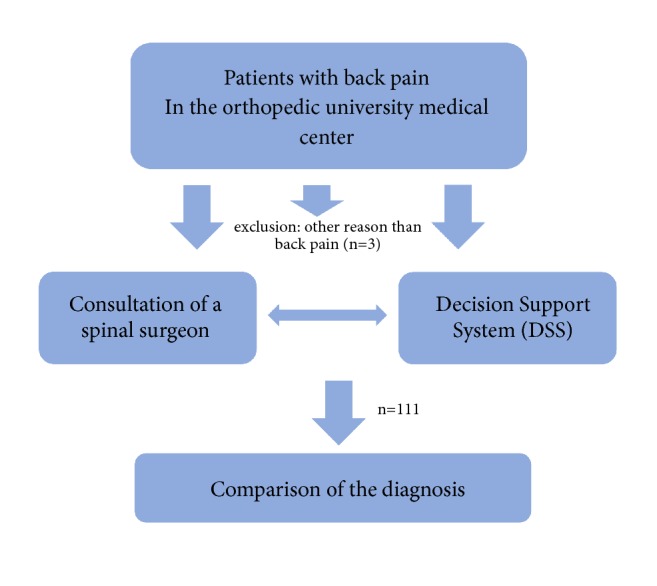
Flowchart of patient inclusion.

The patients were included in the study, when fulfilling the described criteria. From the 114 patients 3 had to be excluded because they did not suffer from back pain as originally supposed. From the remaining 111 patients, 53 were female and 58 males; see Table 1 and Figure 1. There was no significant difference in age between participating female and males, *t*(109) = .113, *p* = .911 (Table 1).

### 3.2. Decision Support System (DSS) Back Pain

The computerized tool of the diagnosis of back pain was developed in cooperation of members of the health insurance Allianz, medical doctors, and psychologists of the University Hospital and University of Regensburg and the Asklepios Clinic Bad Abbach.

Basis for the anamnesis tool algorithm is a scoring table that relates entities (symptoms, patient inherent features, surrogates, etc.) with relevant diagnoses. The strength of the relationship is weighted by literature information on prevalence and severity of the respective diagnosis and the probability of the entities in relation to each diagnosis.

The algorithm starts with a core set of questions on entities, essential to preclassify the back pain and to generate first evidence to preclude red flag diagnoses. Thereby, the algorithm generates a first ranking on the current probability of each diagnosis. For a brief questioning and to generate an intelligent cascade of questions based on the information currently available on the patient, the algorithm generates next-best-questions. From the whole set of remaining questions, the question that differentiates best between the most probable diagnoses is used at each step. The system stops questioning when a most probable diagnosis has elaborated from all other diagnoses.

In a second questioning part, working similar to the diagnosis part, the routing of patients to contacts in the health care system is arranged (self-treatment, specialist, emergency hospitalization, etc.). For a defined set of routing options for each diagnosis, the most appropriate option is again elaborated by fixed questions and next-best-questions. Thereby entities from the diagnosis part already answered and also relevant for the routing part support in differentiating the routing options.

Finally, after the most appropriate combination of diagnosis and contact in the health care system is defined for the patient, each combination of diagnosis and routing option provides a unique therapy recommendation.

### 3.3. Procedure

The study was conducted in at the Department of Orthopaedics of the University Medical Centre Regensburg, Asklepios Medical Centre Bad Abbach. The patients were examined by one of three spine specialists, having a common treatment philosophy. Each examination took between 10 and 20 minutes. The diagnosis with the computer analyses tool took place either before or after the physical examination by an independent examiner in a separate room. The order was chosen according to a smooth integration in the daily clinical routine. The medical doctor was blinded in that way that he did not have any knowledge of the result of the Decision Support System. Thus, both procedures (DSS and medical examination) were treated independently.

### 3.4. Statistical Analysis

The scored diagnoses were given either by the medical doctor manually or by the DSS computerized. At first, the correlation between the diagnoses of the DSS and the medical diagnosis was calculated. Because data were nominal scaled, and both variables have more than two characteristics, Cramers V was used. All diagnoses were described in the result section. Furthermore, the combination between the medical and the computerized diagnoses was presented for every single diagnosis.

Second, the treatment recommendation from the DSS and the medicine were compared. Those recommendations were presented in six possible levels (1= emergency; 2= during the next 24 hours the visit of a specialist is necessary, 3= during the next days the visit of a specialist is necessary, 4= subacute and acute back pain without psychosocial set of problems, 5= subacute back pain with psychosocial set of problems, 6= chronic back pain). Again, Cramer's V was calculated. The different comparisons of the treatment recommendations were classified as possible over- or undertreatment. A possible difference in the frequency was statistically investigated with the Chi-Square test.

## 4. Results

### 4.1. Relation between the DSS and the Diagnosis of the Medical Doctor

The results showed a statistically significant relation between the DSS and the diagnosis of the* medical doctor*,* Cramer's V* = .424,* p* < .001, which can be taken as a medium relationship. This relation holds true, if the data were calculated separately for females,* Cramer's V* = .522,* p* = .020 and males,* Cramer's V* = .504,* p* = .001; see Table 2.

Table 2 shows the highest accordance in the diagnosis of the spinal stenosis. Furthermore, it could be pointed out that the DSS detected six diagnoses, which were not realized from the medical doctors (fibromyalgia, osteoporotic vertebra fracture, cervical distortion, diabetic polyneuropathy, metastasis, arthritis of the hip), whereas the doctors detected three diagnoses which were not detected from the DSS (arthritis, spondylodiscitis, and several singles diagnoses which were combined under the term “others”). The small number of the single combinations does not allow any further statistical analyses.

### 4.2. Relation between the DSS and the Medical Recommendation

There is a significant correlation between the DSS and the medical recommendation,* Cramers V* = .293, *p* = .021, which can be considered as a small to medium effect. In 49.6% of the cases the recommendations were concordant, in 36% they were overestimated, and in 14.4% they were underestimated by the DSS. The frequencies of the categories were statistically significant, *χ*2 (2,* N = 111)* = 20.02,* p *< .001); however, the difference of the frequencies between the categories “overestimation” and “underestimation” was not significant, *χ*2 (1, *N* = 95) = 2.37,* p*= .124). There was one case, where the treatment recommendation of the doctors was an emergency case, which was recognized by the DSS, too.

## 5. Discussion

The study showed a significant relation between the medicine and the DSS; this holds true for the diagnosis as well as the therapeutic recommendation. The result gives a hint that it is possible to use the DSS for a first recommendation. However, the correlational effects are rather small or medium sized so that a continuous development seemed to be reasonable. In general, it was difficult to identify the specific hypotheses consistently, but the identification of the group of the diagnosis was possible. With the help of the DSS, the diagnoses were given from the computer program, so that the patients do not have to choose the correct diagnoses. This is an important advantage, because Bison et al. (2016) showed that only 58% of 328 patients who completed a symptom checker were able to identify the cause of their knee pain when they were given a list of 2 to 15 diagnoses [16].

Due to the fact that the DSS is a program, which should lead to a first treatment recommendation and should not replace the medical doctor, the pilot study showed that the DSS is successful. In future, systems like this DSS should help to prioritize patients before entering the emergency room, to help making decisions on telephone hotline or even to help general practitioners in their decision whether to wait and see or to send to a spinal surgeon immediately. The DSS can be used in waiting rooms as stand-alone tablet or on telephone hotlines by medical staff.

Concerning the therapeutic recommendation, the DSS can be considered as conservative, because the overestimation was more pronounced than the underestimation. This is consistent with the study of Semigran et al. (2015), showing that symptom checkers are more risk-averse [15]. Also, telephone triage can lead to unnecessary care seeking [15, 17]. Those diagnoses, which were underestimated by the DSS, were subacute or chronic back pain. In this case, the doctor has the freedom to choose from a variety of treatments and is led by his personal experience. This is different by the DSS, where the treatment recommendation is given through the same calculation anyway. Due to this, there were deviant treatment recommendations, but these are not hazardous for the patients. Furthermore, it seemed to be worth comparing the results of the DSS with the concordance of telephone triage recommendation and in person-physician recommendation. This concordance ranges from 61% in a study of paediatric abdominal pain to 69% in a multicentre observational study [18, 19]. In future, the system must also be adapted to have recommendation regarding imaging and blood tests in certain indication. For example, the first clinical appearance of a spinal canal stenosis should be first treated conservatively; the second recommendation should be an MRI.

It is recommended to investigate the relation between DSS and the recommendation of the medical doctors while integrating the opinion of orthopaedic surgeons working at outpatient clinics. It is noticeable that there were a minor number of psychiatric diagnoses, a result which is not in line with the literature because depression and anxiety are related to back pain as well as avoidance behavior [15, 20].

Despite the growing use of symptom checkers, this study is the first to use a specific kind of symptom checker, which is based on more than 900 studies and is compared to the diagnoses and recommendations of the medical doctor.

However, the study is first limited of the rather small number of included patients. Second, only spinal surgeons who work in at a hospital were included. In a next step medical doctors working in an outpatient clinic should be consulted, too.

## 6. Conclusion

This pilot study showed good results when using the DSS in patients with low back pain for a first diagnostic decision. Further evaluations and monitoring of the DSS with other diseases will be important to assess the role, which decision support system could play in public health.

## Figures and Tables

**Table 1:** Mean age in years (SD) of participating females and males.

	mean (SD)	min.	max.
female	59.47 (15.81)	25	93
male	59.17 (12.07)	34	83

**Table 2:** Combination of single diagnoses from the DSS and the medicine.

DSS	MEDICINE											
	1	2	3	5	9	10	11	12	14	16	17	Total
1	16	13	2	0	0	10	0	0	1	1	3	46
2	3	4	3	0	0	1	1	0	0	0	2	14
3	1	3	5	0	0	1	0	0	0	0	0	10
4	3	0	1	0	0	0	1	0	0	0	0	5
5	0	1	0	8	1	0	1	1	2	0	1	15
6	1	0	0	1	0	0	2	0	0	0	0	4
7	0	0	0	2	0	0	0	0	0	0	0	2
8	1	0	0	0	0	0	0	0	0	0	0	1
10	0	0	0	0	0	3	0	0	0	0	0	3
11	1	2	1	1	0	1	0	0	0	0	0	6
13	0	0	0	0	0	0	0	0	1	0	0	1
14	0	0	0	1	0	0	0	0	1	0	0	2
15	0	0	1	0	0	0	0	0	0	0	0	1
16	0	1	0	0	0	0	0	0	0	0	0	1

GESAMT	26	24	13	13	1	16	5	1	5	1	6	111

*Legend*. 1=spinal stenosis, 2=unspecific low back pain, 3=Iliosacral joint block, 4= fibromyalgia, 5=cervical/ thoracic myelogelosis 6=osteoporotic vertebra fracture, 7=cervical distortion, 8=diabetic polyneuropathy, 9=facet joint arthritis, 10=lumbar herniated disc, 11=coccygodynia, 12=spondylodiscitis, 13=metastasis, 14=cervical herniated disc, 15= arthritis of the hip, 16=psychological disorder, 17= others.

## Data Availability

The data used to support the findings of this study are available from the corresponding author upon request.

## References

[1] Meucci R. D., Fassa A. G., Faria N. M. (2015). Prevalence of chronic low back pain: systematic review. *Revista de Saúde Pública*.

[2] Melloh M., Röder C., Elfering A. (2008). Differences across health care systems in outcome and cost-utility of surgical and conservative treatment of chronic low back pain: a study protocol. *BMC Musculoskeletal Disorders*.

[3] Kent P. M., Keating J. L. (2005). The epidemiology of low back pain in primary care. *Chiropractic & Osteopathy*.

[4] Vos T. (2016). Global, regional, and national incidence, prevalence, and years lived with disability for 310 diseases and injuries, 1990–2015: a systematic analysis for the Global Burden of Disease Study 2015. *Lancet*.

[5] Benyamin R. M. (2012). The effectiveness of lumbar interlaminar epidural injections in managing chronic low back and lower extremity pain. *Pain Physician*.

[6] Brunner M., Schwarz T., Zeman F., König M., Grifka J., Benditz A. (2018). Efficiency and predictive parameters of outcome of a multimodal pain management concept with spinal injections in patients with low back pain: a retrospective study of 445 patients. *Archives of Orthopaedic and Trauma Surgery*.

[7] Manchikanti L. (2014). Do Cervical Epidural Injections Provide Long-Term Relief in Neck And Upper Extremity Pain? A Systematic Review. *The Spine Journal*.

[8] Muquit S., Ammar A., Nasto L., Moussa A. A., Mehdian H., Vloeberghs M. H. (2016). Combined selective dorsal rhizotomy and scoliosis correction procedure in patients with cerebral palsy. *European Spine Journal*.

[9] DC P. C., DC J. D., Carroll L. (2000). The Factors Associated With Neck Pain and Its Related Disability in the Saskatchewan Population. *The Spine Journal*.

[10] Leboeuf-Yde C., Fejer R., Nielsen J., Kyvik K. O., Hartvigsen J. (2012). Pain in the three spinal regions: the same disorder? Data from a population-based sample of 34,902 Danish adults. *Chiropractic & Manual Therapies*.

[11] Fejer R., Kyvik K. O., Hartvigsen J. (2006). The prevalence of neck pain in the world population: a systematic critical review of the literature. *European Spine Journal*.

[12] Benditz A., Brunner M., Zeman F. (2017). Effectiveness of a multimodal pain management concept for patients with cervical radiculopathy with focus on cervical epidural injections. *Scientific Reports*.

[13] Radhakrishnan K., Litchy W. J., O'Fallon W. M., Kurland L. T. (1994). Epidemiology of cervical radiculopathy: a population-based study from Rochester, Minnesota, 1976 through 1990. *Brain*.

[14] Reuters (2013). *Aetna Brings New Itriage Employer Technology to Mid-Sized Businesses*.

[15] Semigran H. L., Linder J. A., Gidengil C., Mehrotra A. (2015). Evaluation of symptom checkers for self diagnosis and triage: audit study. *BMJ*.

[16] Bisson L. J., Komm J., Bernas G. A., Marzo J. M., Rauh M. A., Browning W. M. (2015). How Accurate Are Patients At Diagnosing The Cause Of Their Knee Pain With The Help Of A Web-based Symptom Checker?. *Orthopaedic Journal of Sports Medicine*.

[17] Poote A. E., French D. P., Dale J., Powell J. A. (2014). A study of automated self-assessment in a primary care student health centre setting. *Journal of Telemedicine and Telecare*.

[18] Staub G. M., von Overbeck J., Blozik E. (2013). Teleconsultation in children with abdominal pain: a comparison of physician triage recommendations and an established paediatric telephone triage protocol. *BMC Medical Informatics and Decision Making*.

[19] Giesen P., Ferwerda R., Tijssen R. (2007). Safety of telephone triage in general practitioner cooperatives: do triage nurses correctly estimate urgency?. *Quality & Safety in Health Care*.

[20] Wertli M. M., Rasmussen-Barr E., Weiser S., Bachmann L. M., Brunner F. (2014). The role of fear avoidance beliefs as a prognostic factor for outcome in patients with nonspecific low back pain: a systematic review. *The Spine Journal*.

